# Eosinophil cationic protein as a marker for assessing the efficacy of tacrolimus ophthalmic solution in the treatment of atopic keratoconjunctivitis

**Published:** 2011-04-13

**Authors:** Tais Hitomi Wakamatsu, Mari Tanaka, Yoshiyuki Satake, Murat Dogru, Kazumi Fukagawa, Ayako Igarashi, Hiroshi Fujishima

**Affiliations:** 1 Johnson & Johnson Department of Ocular Surface and Visual Optics, Keio University School of Medicine, Tokyo, Japan; 2 Department of Ophthalmology, Keio University School of Medicine, Tokyo, Japan; 3 Yatsu Ekimae Ajisai Eye Clinic, Chiba, Japan; 4 Department of Ophthalmology, Tokyo Dental College Ichikawa Hospital, Chiba, Japan; 5 Department of Ophthalmology, Tsurumi University School of Dental Medicine, Kanagawa, Japan

## Abstract

**Purpose:**

To examine the clinical efficacy and anti–inflammatory effects of tacrolimus eye drops; we studied the changes in clinical ocular findings and measured tear eosinophil cationic protein (ECP) levels of atopic keratoconjunctivitis (AKC) patients before and after the treatment.

**Methods:**

Nine eyes of 9 patients (8 males, 1 female; mean age: 16.9±11.4 years; range: 6–44 years) diagnosed with moderate or severe AKC disease were enrolled in this prospective study and treated with tacrolimus. All patients received 0.1% tacrolimus eye drops 2 times a day for 1 month. Tear samples were taken before and after treatment and ECP concentrations were obtained. Corneal fluorescein staining and conjunctival injection, edema, and papillary formation were graded on the recruitment day and one month later. Analysis of pre- and post-treatment findings was done using the Wilcoxon signed test. The ECP concentrations were correlated with the clinical signs using Spearman correlation tests.

**Results:**

Post-treatment tear ECP levels were significantly reduced compared to the pre-treatment level. Clinical corneal scores also improved significantly after one month treatment with tacrolimus eye-drops. The mean conjunctival injection and conjunctival edema scores were significantly (p<0.05) decreased after the drug therapy. Strong positive linear correlations between ECP values and clinical signs were observed. Patients did not present side effects during the treatment with tacrolimus.

**Conclusions:**

In this pilot study, tacrolimus eye drops were found to reduce signs of AKC. ECP proved to correlate well with clinical signs of AKC.

## Introduction

Atopic keratoconjunctivitis (AKC) is a chronic atopic disease of the conjunctiva and cornea. The disease occurs in both children and adults and is associated with atopic dermatitis (AD). The male to female ratio varies from 2.4:1 to below 1:1 depending on the report [1-3]. AKC is the most debilitating of the allergic conjunctival diseases, owing to its chronicity and ability to cause loss of vision due to frequent corneal complications [1,4,5]. Ocular symptoms include intense itching, photophobia, burning, and foreign body sensation. The clinical signs are observed as corneal staining, conjunctival injection, edema, and papillae on the upper tarsal conjunctiva. In the most severe cases, ulceration and neovascularization of the cornea, subepithelial fibrosis of the conjunctiva, fornix shortening, and symblepharon are present. Eosinophils, mast cells, neutrophils and lymphocytes, specially activated T-cells, are seen in conjunctival cytology as an inflammatory response of the atopic disease [6-8]. We previously reported the presence of neutrophils and dendritic cells in conjunctival epithelium and papillary formations visualized by in vivo confocal scanning microscopy of the upper palpebral conjunctiva [9]. Owing to the complexity, chronicity and multifactorial nature of the AKC disease, no evidence exists showing a significant therapeutic effect on the disease course. The goals of treatment are to achieve symptomatic control, reduce the frequency of corneal complications and their morbidity, and to minimize the side effects of treatment.

Treatment of AKC based on eye-drops containing antihistamines or sodium chromoglycate and its derivates are often insufficient [10,11]. The addition of steroids is usually mandatory. Although these agents often achieve symptomatic and inflammatory control in a short time of treatment, they present disadvantages well recognized as their side effects, including glaucoma, cataract, herpes simplex virus keratitis, and atrophy of the derma of the eyelids [12] that limit its use to short courses, resulting in inadequate long-term treatment responses. As an alternative to steroids, several immunosuppressive medications have been considered. Cyclosporine eye drops, tacrolimus ointment and most recently tacrolimus eye drops have been released for use in ocular allergy [13-15].

Tacrolimus (FK-506) is a strong immunosuppressant that inhibits the proliferative response of lymphocytes to alloantigen stimulation and a variety of T cell associated immune reactions. It has been isolated from the fermentation broth of *Streptomyces tsukubaenis* as colorless prism and the molecular formula was determined as C44H69NO12.H2O. Tacrolimus suppresses the immune responses by inhibiting the inflammatory cytokine release (e.g., interleukin-2, IL-3, IL-4, IL-5, IL-8, interferon-gamma, tumor necrosis factor-α) and also down-regulates the high-affinity IgE receptor I (FcRI) expression on Langerhans cells [16-20] without adversely affecting connective tissue [21]. It has been shown to be a potent immunosuppressive agent in vivo and in vitro. Tacrolimus shares several immunosuppressive properties with cyclosporine A, although it is known to be 10 to 100 times more potent in this regard [22,23]. Its safety and efficacy in the treatment of atopic dermatitis have been demonstrated in short- and long-term studies with adult and pediatric patients [17-19,24,25]. Previous studies on the use of tacrolimus ointment in atopic eyelid disease have also shown good levels of response and improvement in conjunctivitis symptoms, with no significant adverse events [26,27].

Eosinophil cationic protein (ECP) is one of several highly basic proteins present in the secretory granule of the eosinophil that is released upon its activation. ECP has already been found to play an important role in the pathogenesis of allergic conjunctivitis and tear ECP levels in allergic patients are known to be related to the severity of clinical findings [28-31].

The purpose of this study was to examine the clinical efficacy and anti-inflammatory effects of tacrolimus eye drops, evaluating the changes of clinical ocular findings and measuring tear ECP levels of AKC patients before and after treatment.

## Methods

### Subjects

This study was conducted at the Ocular Allergy Subspecialty Clinic of the Department of Ophthalmology, Mita Hospital, International University of Health and Welfare (Tokyo, Japan). The study protocol was approved by the Ethical Committee of Mita Hospital, International University of Health and Welfare review board. This study adhered to the tenets of the Declaration of Helsinki. The clinical trial registration number is UMIN000001262.

Nine eyes of 9 AKC patients (8 males, 1 female) aged between 6 and 44 years (mean age: 16.9 years) were recruited sequentially from June 2008 to February 2010 in a prospective study. All patients were known to have had a long-standing AKC that was previously treated with steroids eye-drops. Patients were selected based on the criteria established by Hogan [32]: chronic conjunctivitis and progressive keratitis in association with atopic keratoconjunctivitis, and presence of a hereditary allergic tendency. All patients had moderate to severe AKC disease with presence of cobblestone-like papillae, severe injection and edema of the upper tarsal conjunctiva and moderate to severe corneal staining scores or ulcers as markers of severe activity and inflammation. Some of them presented elevation of the intraocular pressure due to previous steroid treatment or were refractory to the standard steroid regime. None of the patients had a history of Stevens-Johnson syndrome, chemical, thermal, radiation injury, bacterial, viral or toxic conjunctivitis, or underwent any ocular surgery that would create an ocular surface problem. Subjects in this study did not have a history of contact lens use. Any patient was being treated with systemic cytotoxic immunosuppressants, steroids and prostaglandin inhibitors.

### Procedures and visits

Patients received 0.1% tacrolimus eye drops (Senju Pharma Inc., Osaka, Japan) 2 times a day for 1 month. As the ethic board committee did not allow a washout period in subjects with an active disease to study the naïve ocular surface status, all the anti-allergic and anti-inflammatory medications that the subjects were using on the recruitment day were discontinued and the new medication was started on the next day (day one of the treatment). The administration of other topically drugs except lubricant was not allowed during the study period.

Patients attended several follow-up visits, considering that some of them had corneal erosion or ulcer and underwent tear collection, slit-lamp examination including fluorescein staining, conjunctival injection, and edema and papillae formation grading before and after 1 month of treatment. Venus peripheral blood was collected and enzyme-linked immunosorbent assay (ELISA) test was used to detect the specific IgE antibodies to 26 allergens was performed using the MAST 26 Allergen Kit in all subjects (SRL, Tokyo, Japan).

### Slit lamp examination

A conventional slit-lamp microscopic examination was performed. Two microliters of preservative-free 1% fluorescein preservative-free solution was instilled in the conjunctival sac with a micropipette. The subjects were then instructed to blink several times for a few seconds to ensure adequate covering of the dye onto the ocular surface. Fluorescein staining of the cornea was noted and scored. The cornea was divided into 3 equal upper, middle, and lower zones. Each zone had a staining score ranging between 0 and 3 points, with the minimum and maximum total staining scores ranging between 0 to 9 points. Likewise, the presence of scarce staining in zone 1 was scored as 1 point, whereas punctate staining covering the entire zone was scored as 3 points. A score >3 points was regarded as abnormal [33,34].

Three major upper tarsal conjunctival findings such as conjunctival injection, edema, and papillary formation were assessed, and a clinical severity score [35] was assigned for each finding. The severity of the signs was graded as follows: score 0-absence of signs; score 1-mild; score 2-moderate and 3-severe conjunctival injection, edema or papillary formation.

### Tear analysis

Tear collection was performed before the treatment and during the first month of treatment with tacrolimus. With the use of a capillary micropipette, tears were gently collected from the external canthus, taking precaution to avoid reflex tearing. Tear samples were collected and then patients were asked to wait at least 30 min before the vital staining examination. Following collection, tears were placed in Eppendorff tubes and centrifuged at 13,600× g for 5 min at 4 °C. The supernatants were then stored at −80 °C until assayed.

A commercially available ECP ELISA kit (MBL, Tokyo, Japan) was used to determine the ECP concentration, according to the product protocol. We also investigated the correlation between tear ECP levels and cornea fluorescein staining scores, conjunctival injection, edema, as well as conjunctival papillary proliferation.

### Statistical analysis

The Wilcoxon signed rank test was used to compare the baseline values with the post-treatment values. Spearman correlation test was used to explore various associations: ECP concentration and fluorescein scores, ECP concentration and conjunctival injection, ECP concentration and papillary proliferation and ECP concentration and conjunctival edema. Data were processed using Instat, GraphPad software version Instat 3.0 (San Diego, CA).

## Results

### Patient characteristics

Overall, the disease activity in the study group was moderate to severe. All 9 patients completed the study. Compliance and documentation were generally satisfactory. There were no complains of burning or heat sensation, the most common adverse effects of the tacrolimus ointments. None of the patients presented episode of herpetic keratitis during the treatment with tacrolimus. No significant changes in the intraocular pressure, cornea, lens, refraction or anterior chamber occurred in any patients during the treatment with topical tacrolimus.

### Effect on clinical signs and ocular surface vital staining scores

The mean fluorescein staining scores were 5.50±4.14 points and 0.28±0.75 points in AKC patients before and after treatment with tacrolimus eye-drops, respectively. We could observe a statistically significant difference between the pre- and post-treatment values (p<0.05) as shown in Table 1.

**Table 1:** Comparison of clinical signs and ocular surface vital staining scores before and after treatment with tacrolimus

**Clinical signs and ocular surface vital staining scores**	**Before treatment**	**After 1 month of treatment**	**p value**
Conjunctival injection score(0–3 points)	2.89±0.33	1.00±0.53	0.004
Conjunctival edema score (0–3 points)	2.78±0.67	1.00±0.92	0.016
Papillary proliferation score (0–3 points)	3.00±0.01	2.62±1.06	0.500
Fluorescein staining (0–9 points)	5.50±4.14	0.28±0.75	0.016

**Figure 1:**
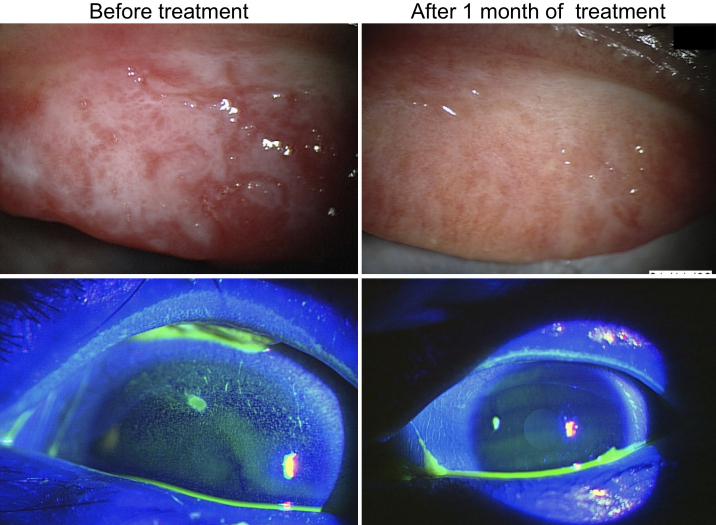
Representative anterior segment photographs from an AKC patient before and after treatment with tacrolimus ophthalmic solution. Anterior segment photograph (right) shows an extensive corneal damage visualized by the fluorescein staining. Note that the superficial punctate keratopathy is present in almost all the surface of the cornea and is associated to the increased papillary formation. The photographs on the left side represent the cornea and papillary formation from the same patient after 1 month of treatment with 0.1% tacrolimus ophthalmic solution. Note the improvement of the corneal damage as well as the decrease of papillary formation and conjunctival inflammatory status.

The pre-treatment mean conjunctival injection score was 2.89±0.33. After drug therapy, the mean conjunctival injection score was significantly (p<0.05) decreased to 1.00±0.53. The pre-treatment mean conjunctival edema score was 2.78±0.67, and after 1 month of treatment, 1.00±0.92. The clinical conjunctival edema score was significantly decreased (p<0.05) after therapy, as shown in Table 1. A representative case of improvement in clinical findings after 1 month of treatment with tacrolimus is shown in Figure 1.

The mean papillary formation score was not significantly different after treatment (2.62±1.06) when compared with the mean value before treatment with tacrolimus (3.00±0.01; p=0.500, Table 1).

### Tear ECP concentrations

**Figure 2:**
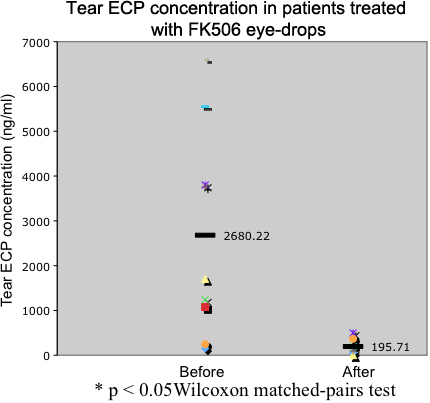
Comparison of tear ECP levels before and after treatment with 0.1% tacrolimus eye-drops. Note the significant decrease in the ECP values in patients treated with tacrolimus eye-drops for 1 month.

The mean pre and post-treatment tear ECP concentrations were 2,680.22±2,342.7 ng/ml and 195.71±164.46 ng/ml, respectively. A significantly difference in relation to the pre- and post-treatment tear ECP concentrations was observed (p<0.05; Figure 2).

**Figure 3:**
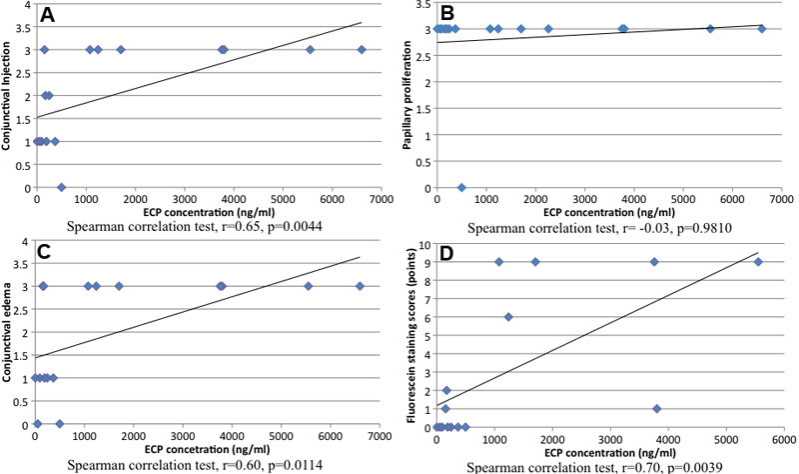
Correlation between conjunctival injection, conjunctival edema, papillary formation, corneal epithelial cell damage and ECP levels. ECP concentrations showed a significant correlation with conjunctival injection scores (**A**: r=0.65, p=0.0044). ECP concentrations did not show any correlation with papillary proliferation scores (**B**: r=-0.03, p=0.9810). ECP concentrations showed a significant correlation with conjunctival edema scores (**C**: r=0.60, p=0.0114).  ECP concentrations showed a significant correlation with fluorescein staining scores (**D**: r=0.70, p=0.0039).

We observed a significant positive correlation between tear ECP concentrations with corneal fluorescein (r=0.70, p=0.0039), conjunctival injection (r=0.65, p=0.0044) and edema scores (r=0.60, p=0.0114), as shown in Figure 3.

## Discussion

The results of our pilot study demonstrate that tacrolimus was effective in reducing signs of AKC in 9 subjects over a month of period.

Previous studies has shown that tacrolimus 0.3% eyedrops can inhibit the infiltration of eosinophils and lymphocytes significantly in experimental animal models [36]. In another study, 0.1%–1% tacrolimus eyedrops inhibited the late and delayed-type inflammatory response of experimental animal allergic conjunctivitis with an efficacy similar to that of betamethasone 0.1% eyedrops [37]. Tacrolimus is a very hydrophobic macrolide lactone with a molecular weight of approximately 800 daltons. Because of its characteristics and relatively large molecular size, it should penetrate the corneal epithelium with some difficulty and accumulate in the cornea stroma. This may result in low intraocular drug levels [38]. However, when the corneal epithelial barrier is broken it certainly penetrates easier than over an intact ocular surface which may confer successful treatment in the severe cases. Because of these drug characteristics, patients with conjunctival inflammatory conditions should theoretically respond better to topical tacrolimus than patients with inflammatory conditions such as penetrating keratoplasty rejection and uveitis.

The papillary formation score did not show significant difference. We thought one month period of treatment was not sufficient to observe significant changes in the improvement of papilla formation. Moreover, some cases in 9 patients were longstanding cases of AKC and once fibrosis in papilla formation was established the medication affected less significant.

In our study, we observed significantly decrease in the ECP values after 1 month of treatment with tacrolimus. Furthermore, strong positive linear correlations between ECP values and clinical signs were observed. By measuring ECP levels in tears of patients with allergic conjunctivitis, one can estimate the degree of inflammation and may also assess the correlation of the mediators to the severity of the clinical findings. The measurement of ECP levels seems to be one potent marker to do the follow-up of allergic conjunctivitis and evaluate the responsiveness to a specific treatment.

The main findings of this study suggest that 0.1% tacrolimus ophthalmic solution offers an efficient option for the treatment of severe AKC, although this pilot group is based in a small number of subjects and the course of the treatment is maybe considered short. The measurement of ECP demonstrated to be an important marker in the diagnosis and monitoring of the atopic disease, and also may prove to be useful tool for the evaluation of new therapies for the AKC disease.

It remains the further goal of future studies to determine how are the changes of inflammatory cytokines and markers of inflammation in response of treatment with tacrolimus. Such investigations will give insight into the underlying mechanisms of tacrolimus and atopic keratoconjunctivitis.
